# The value of whole-volume apparent diffusion coefficient histogram analysis in preoperatively distinguishing intracranial solitary fibrous tumor and transitional meningioma

**DOI:** 10.3389/fonc.2023.1155162

**Published:** 2023-05-16

**Authors:** Gang Wang, Junlin Zhou

**Affiliations:** ^1^ Department of Radiology, The First Hospital of Lanzhou University, Lanzhou, China; ^2^ Key Laboratory of Medical Imaging of Gansu Province, Lanzhou, China; ^3^ Department of Radiology, Lanzhou University Second Hospital, Lanzhou, China; ^4^ Gansu International Scientific and Technological Cooperation Base of Medical Imaging Artificial Intelligence, Lanzhou, China

**Keywords:** solitary fibrous tumor, transitional meningioma, magnetic resonance imaging, apparent diffusion coefficient, histogram analysis

## Abstract

**Purpose:**

To investigate the value of whole-volume apparent diffusion coefficient (ADC) histogram analysis in preoperatively distinguishing intracranial solitary fibrous tumors (SFT) from transitional meningiomas (TM), thereby assisting the establishment of the treatment protocol.

**Methods:**

Preoperative diffusion-weighted imaging datasets of 24 patients with SFT and 28 patients with TM were used to extract whole-volume ADC histogram parameters, including variance, skewness, kurtosis, and mean, as well as 1st (AP1), 10th (AP10), 50th (AP50), 90th (AP90), and 99th (AP99) percentiles of ADC using MaZda software. The independent *t*-test or Mann–Whitney *U* test was used to compare the differences between ADC histogram parameters of SFT and TM. Receiver operating characteristic (ROC) curves were generated to evaluate the performance of significant ADC histogram parameters. Spearman’s correlation coefficients were calculated to evaluate correlations between these parameters and the Ki-67 expression levels.

**Results:**

SFT exhibited significantly higher variance, and lower AP1 and AP10 (all *P* < 0.05) than TM. The best diagnostic performance was obtained by variance, with an area under the ROC curve of 0.848 (0.722–0.933). However, there was no significant difference in skewness, kurtosis, mean, or other percentiles of ADC between the two groups (all *P* > 0.05). Significant correlations were also observed between the Ki-67 proliferation index and variance (*r* = 0.519), AP1 (*r* = -0.425), and AP10 (*r* = -0.372) (all *P* < 0.05).

**Conclusion:**

Whole-volume ADC histogram analysis is a feasible tool for non-invasive preoperative discrimination between intracranial SFT and TM, with variance being the most promising prospective parameter.

## Introduction

Intracranial solitary fibrous tumors (SFT) are a rare type of neoplasm of meningeal mesenchymal cell origin (1, 2). Transitional meningiomas (TM) originate from meningeal arachnoid cells and are one of the most common subtypes of meningioma (3, 4). Pathologically, TM present with transitional morphological characteristic between that of endothelial and fibrous meningioma (4, 5). In clinical practice, SFT and TM have similar clinical manifestations and conventional imaging characteristics, which makes preoperative discrimination challenging. However, there are significant differences between the biological behavior, treatment options, and prognosis of the two tumors (1, 4). Compared with TM, SFT exhibit more aggressive biological behavior and are prone to recurrence and distant metastases after surgery (6). Surgical resection, combined with radiotherapy or chemotherapy, is the recommended treatment modality (7). In addition, the risk of intraoperative bleeding is significantly greater owing to the abundant blood supply of SFT (8). Given the above consideration, accurate preoperative differentiation between SFT and TM is therefore of great importance for establishing clinical treatment protocols.

Diffusion-weighted imaging (DWI) enables non-invasive assessment of the tumor microenvironment characteristics by examining the movement of water and is quantified by apparent diffusion coefficient (ADC) (9, 10). However, due to tumor heterogeneity, ADC values obtained from the region of interest (ROI) of the local lesion do not fully reflect the overall characteristics of the tumor (11). Whole-volume ADC histogram analysis, an objective and reproducible image processing technique, can provide more information about tumor characteristics, and is an effective method for a comprehensive assessment of tumor heterogeneity (12, 13). Recently, several studies have shown that ADC histogram analysis has great application potential and clinical value in the diagnosis, differentiation, and prognostic assessment of meningeal tumors (2, 13, 14).

To date, no published studies have investigated the use of ADC histogram analysis to distinguish between SFT and TM. Therefore, this study aimed to investigate the value of whole-volume ADC histogram analysis in preoperatively distinguishing between SFT and TM. In addition, we further assessed the correlation between significant ADC histogram parameters and Ki-67 expression levels.

## Materials and methods

### Patients

This retrospective, single-center study was approved by our institutional review board, and the requirement for informed consent was waived for all patients. From January 2017 to October 2022, twenty-four patients with intracranial SFT and 28 patients with TM were enrolled in this study. The inclusion criteria were as follows: (1) patients with defined pathologically diagnosed after operation; (2) patients underwent standard preoperative brain magnetic resonance imaging (MRI), including DWI sequences. The exclusion criteria were as follows: (1) any clinical treatment before MRI examination; (2) poor ADC image quality not meeting the needs of histogram analysis. This study has been reported by the Standards for Reporting Diagnostic Accuracy Studies (STARD) checklist.

### MRI acquisition

MRI images were obtained using a 3.0 T MR scanner (Siemens Verio, Erlangen, Germany). The unenhanced scan sequences included axial T1-weighted imaging (WI) (repetition time [TR], 250 ms; echo time [TE], 2.48 ms) and axial T2WI (TR 4000 ms; TE 96 ms), with a 220 mm×220 mm field of view (FOV), 5 mm slice thickness, 1.0 mm interslice gap, and a 256 × 256 matrix. DWI used the echo planar-imaging sequence plus frequency selective fat suppression technology, with TR 4500 ms, TE 102 ms, 220 mm×220 mm FOV, 5 mm slice thickness, 1.0 mm interslice gap, 256 × 256 matrix, and b-values of 0 and 1,000 s/mm2. Contrast-enhanced T1WI (CET1) scanning was performed using Gd-DTPA (Bayer Schering Pharma AG, Berlin, Germany) as the contrast agent, intravenously administered as a bolus injection of 0.1 mmol/kg at a flow rate of 3.0 mL/s. Finally, T1WI, T2WI, DWI, and CET1 scans were obtained for each patient before treatment.

### Image analysis

MaZda software (version 4.7, The Technical University of Lodz, Institute of Electronics, http://www.eletel.p.lodz.pl/mazda/) was used to conduct the ADC histogram analysis. Two experienced neuroradiologists (XX and XXX, with 25 and 7 years of brain MRI experience, respectively), blinded to the histopathological results, independently reviewed the MR images. During the histogram analysis, T1WI, T2WI, and CET1 were used to determine the tumor boundary. Firstly, the ROI containing all components of the tumor was sketched on the T2WI image, and the ROIs were then copied to the ADC maps for conducting histogram analysis. Finally, the software automatically calculated and extracted nine histogram parameters, including variance, skewness, kurtosis, and mean, as well as 1st (AP1), 10th (AP10), 50th (AP50), 90th (AP90), and 99th (AP99) percentiles of ADC (14).

### Statistical analysis

All data analysis were performed using MedCalc software (v.19.1, Mariakerke, Belgium). The chi-square test was used to compare categorical variables. The inter-observer reliability of the ADC histogram parameters was evaluated using the intraclass correlation coefficient (ICC). Continuous variables were presented as mean ± standard deviation (normal distribution) or median (lower quartile, upper quartile) (non-normal distribution), and compared using the independent *t*-test or Mann–Whitney *U* test, respectively. The performance of significant ADC histogram parameters was evaluated by generating receiver operating characteristic (ROC) curves and calculating the area under the curve (AUC), sensitivity, specificity, positive predictive value, negative predictive value, and accuracy. Spearman’s correlation coefficients were calculated to evaluate the correlations between these parameters and the Ki-67 expression levels. In addition, Bonferroni correction was used for multiple testing, and the Delong test was used to compare the difference between AUCs. *P*-values< 0.05 were regarded as statistical significance.

## Results

Twenty-four patients with SFT (mean age, 49.42 ± 14.86; range, 11–74 years) and 28 patients with TM (mean age, 50.86 ± 11.49; range, 18–70 years) were enrolled in this study. The SFT group included 14 female and 10 male patients, whereas the TM group included 17 female and 11 male patients. However, there were no significant differences in age (*P* = 0.695) or sex (*P* = 0.862) between the SFT and TM groups.

**Figure 1:**
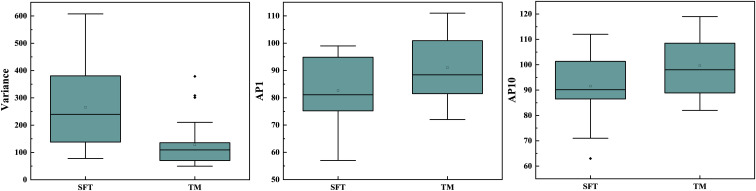
Box plot displaying the difference in variance, AP1, and AP10 between SFT and TM.

**Figure 2:**
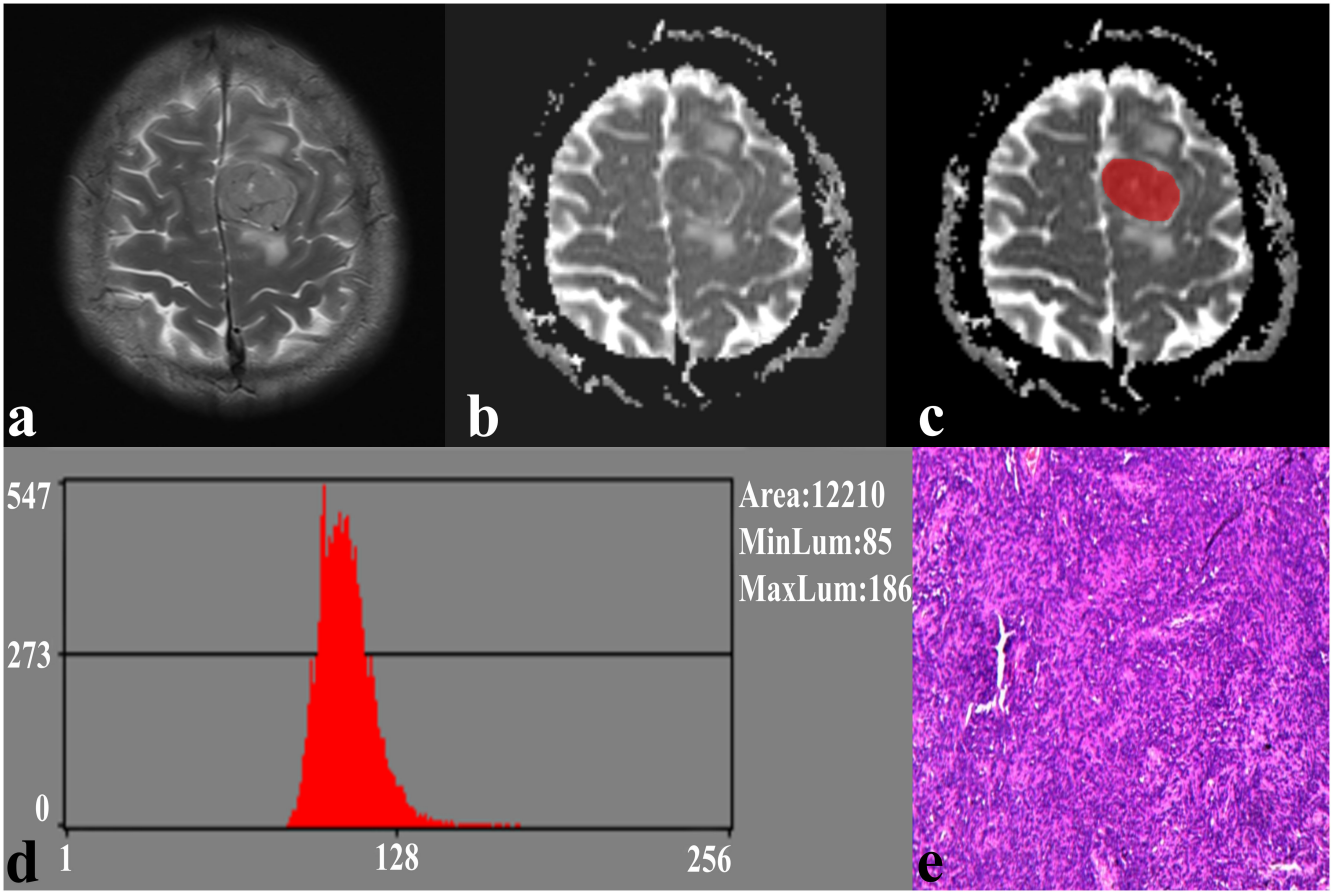
A 41-year-old male with SFT. **(A)** The axial T2WI image shows an inhomogeneous lesion on the left side of the falx cerebri. **(B)** The lesion shows an uneven low signal on the ADC image. **(C)** The lesion covered ROI on the ADC image. **(D)** Histogram of the ROI. **(E)** Pathological analysis confirms SFT (hematoxylin and eosin, ×100).

**Figure 3:**
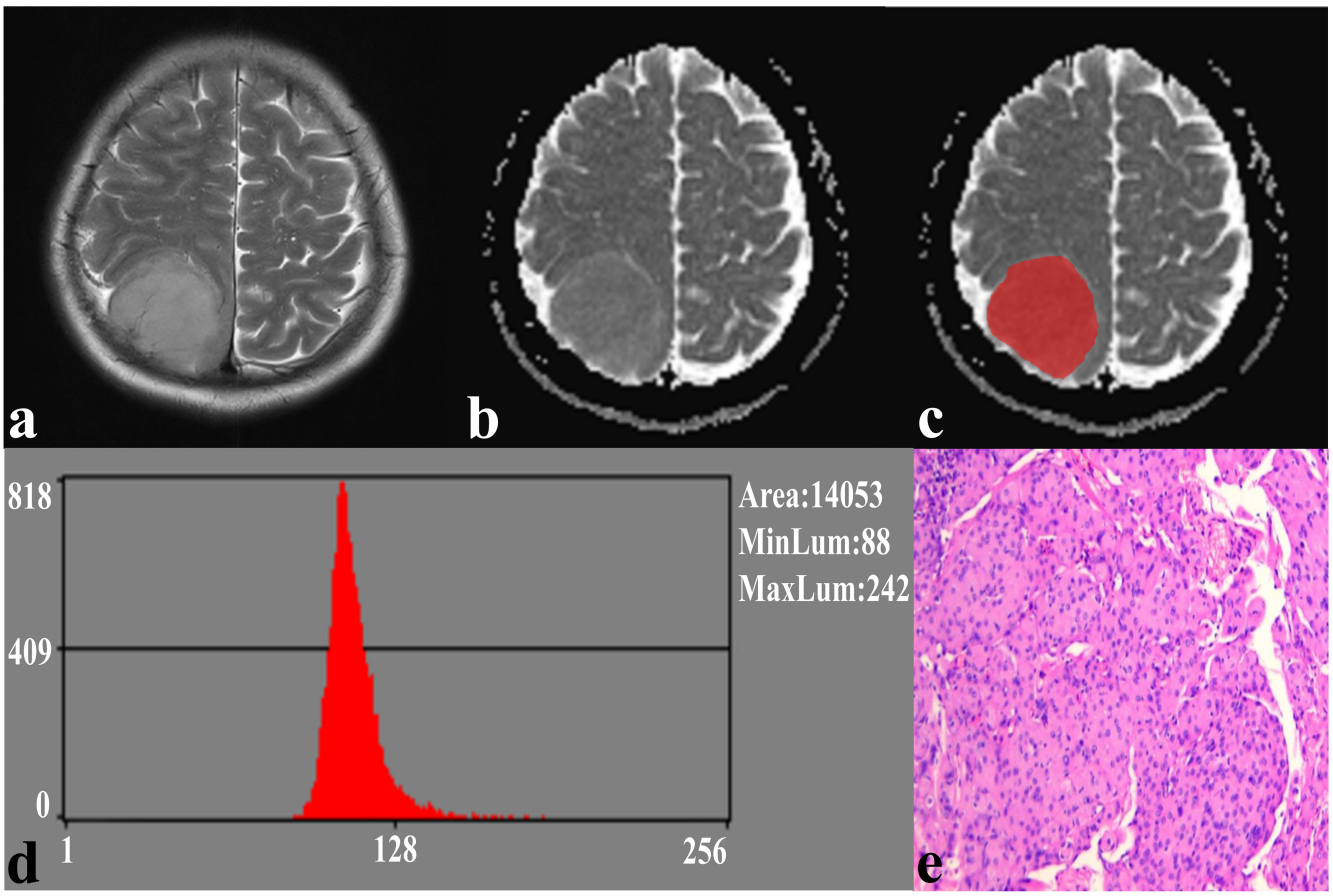
A 59-year-old female with TM. **(A)** The axial T2WI image shows a homogeneous, well-defined lesion on the right parietal region. **(B)** The lesion shows a low signal on the ADC image. **(C)** The lesion covered ROI on the ADC image. **(D)** Histogram of the ROI. **(E)** Pathological analysis confirms TM (hematoxylin and eosin, ×100).

In the repeatability analysis, all histogram parameters exhibited excellent inter-observer agreement (ICC: 0.795–0.932, **Table 1**). SFT showed significantly higher variance, and lower AP1 and AP10 (all *P* < 0.05), compared to TM (**Figure 1**). However, there was no significant difference in skewness (*P* = 0.995), kurtosis (*P* = 0.091), mean (*P* = 0.069), AP50 (*P* = 0.637), AP90 (*P* = 0.548), or AP99 (*P* = 0.257) between the two groups (**Table 1**). Representative cases of SFT and TM are shown in **Figures 2** and **3**, respectively.

**Table 1:** Comparison of ADC histogram parameters between SFT and TM.

Parameters	SFT (n=24)	TM (n=28)	*P* value	ICC (95%CI)
Variance	239.59 (137.82, 383.51)	109.51 (67.13, 137.59)	*P*<0.001	0.932 (0.885, 0.961)
Skewness	0.86 ± 0.68	0.86 ± 0.65	0.995	0.843 (0.742, 0.907)
Kurtosis	1.79 (0.72, 4.73)	0.90 (0.11, 3.11)	0.091	0.795 (0.667, 0.877)
Mean	109.53 (103.61, 114.20)	116.01 (107.22, 119.52)	0.069	0.828 (0.719, 0.898)
AP1	82.63 ± 12.84	91.00 ± 10.63	0.013	0.881 (0.901, 0.930)
AP10	91.58 ± 12.36	99.64 ± 11.17	0.017	0.892 (0.819, 0.937)
AP50	109.09 ± 11.30	110.74 ± 13.43	0.637	0.855 (0.638, 0.864)
AP90	125.59 ± 11.82	127.84 ± 14.57	0.548	0.817 (0.760, 0.914)
AP99	146.79 ± 17.86	152.45 ± 17.62	0.257	0.846 (0.746, 0.905)

ADC, apparent diffusion coefficient; SFT, solitary fibrous tumor; TM, transitional Meningioma; ICC, intraclass correlation coefficient; CI, confidence interval.

**Figure 4:**
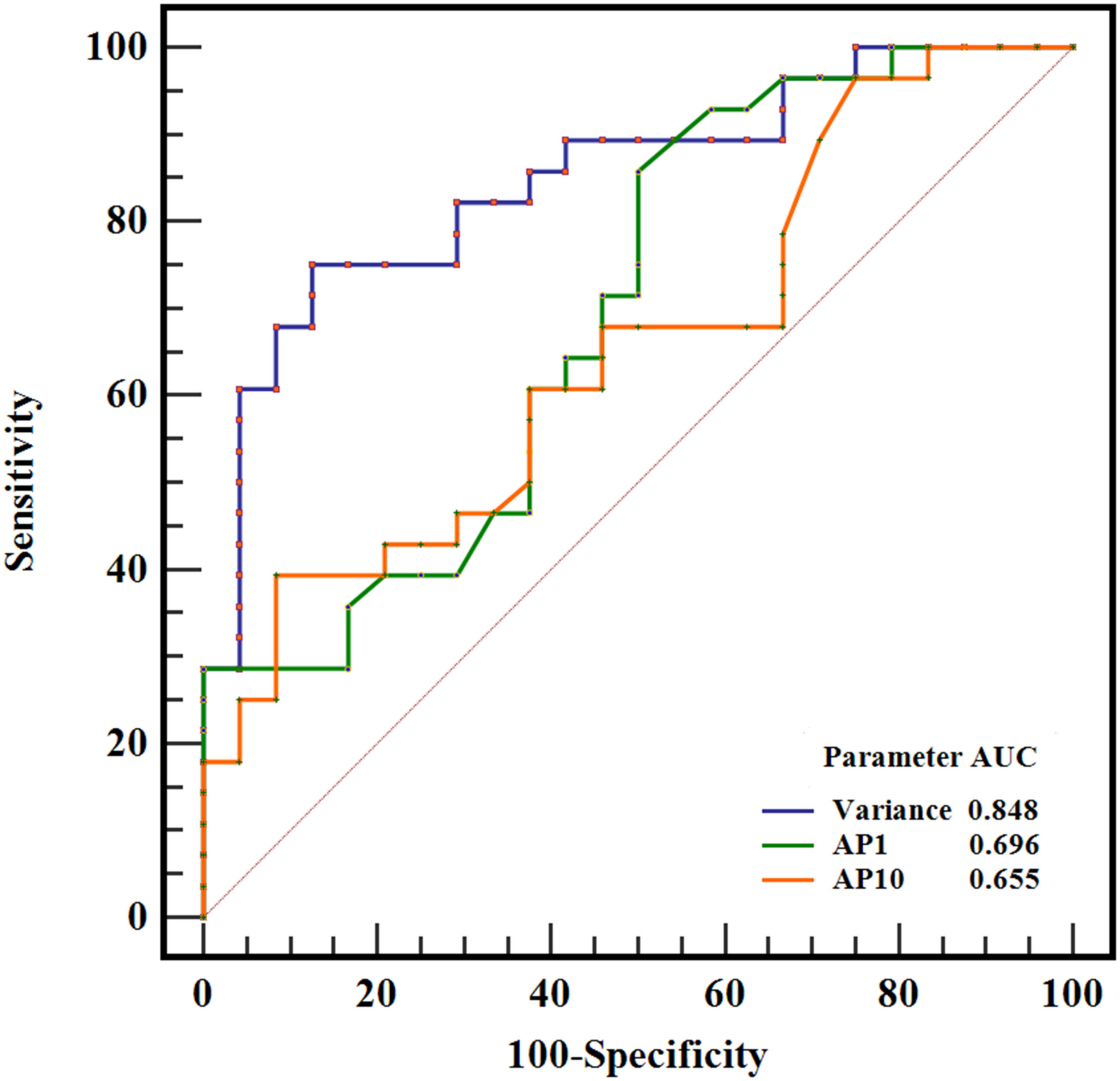
ROC curves of variance, AP1, and AP10 in differentiating SFT and TM, and the variance generated the highest AUC of 0.848.

The results of the ROC curve analysis of the significant ADC histogram parameters are presented in **Table 2** and **Figure 4**. Variance, AP1, and AP10 could effectively distinguish between SFT and TM, with the best diagnostic performance was obtained by variance, with an AUC of 0.848 (0.722, 0.933).

**Table 2:** Diagnostic performance of ADC histogram parameters in distinguishing SFT and TM.

Parameters	AUC (95% CI)	Cutt-off	SE (%)	SP(%)	PP(%)	NP(%)	AC(%)
Variance	0.848 (0.722, 0.933)	131.53	75.00	87.50	87.50	75.00	80.77
AP1	0.696 (0.553, 0.816)	80.00	85.71	50.00	66.70	75.00	69.23
AP10	0.655 (0.510, 0.781)	104.00	39.29	91.67	84.60	56.40	63.46

ADC, apparent diffusion coefficient; SFT, solitary fibrous tumor; TM, Transitional Meningioma; CI, confidence interval; AUC, area under the receiver operating characteristic curve; SE, sensitivity; SP, specificity; AC, accuracy; PP, positive predictive value; NP, negative predictive value.

The Ki-67 expression level data were available for 52 patients and showed a greater Ki-67 proliferation index for the SFT group than for the TM group [(15.58 ± 12.76)% vs (3.61 ± 2.30)%, *P*<0.05]. Significant correlations were observed between the Ki-67 proliferation index and variance (*r* = 0.519), AP1 (*r* = -0.425), and AP10 (*r* = -0.372) (all *P* < 0.05) (**Table 3**).

**Table 3:** Correlations between ADC histogram parameters and the Ki-67 expression.

Parameters	Ki-67 proliferation index	
	*r*	*P*
Variance	0.519	*P*<0.001
Skewness	-0.205	0.145
Kurtosis	0.003	0.982
Mean	-0.214	0.128
AP1	-0.425	0.002
AP10	-0.372	0.007
AP50	-0.143	0.312
AP90	-0.106	0.454
AP99	-0.067	0.636

ADC, apparent diffusion coefficient; *r*, Pearson correlation coefficient.

## Discussion

To our knowledge, no published studies have focused on SFT and TM differentiation using ADC histogram analysis. We demonstrated that whole-volume ADC histogram analysis can differentiate intracranial SFT and TM preoperatively, with a significant correlation observed between variance, AP1, AP10, and the Ki-67 expression. The variance exhibited the best performance in differentiating SFT from TM.

Histogram analysis provides quantitative parameters of tumor heterogeneity by assessing the spatial distribution of voxel levels on medical images (15). It has been reported that the reproducibility and diagnostic performance of histogram parameters are significantly better than higher-order texture parameters for functional MRI images, caused by the lower spatial resolution (16), which is reflected by the excellent inter-observer agreements observed in all ADC histogram parameters in this study. We found that SFT exhibits significantly higher variance, and lower AP1 and AP10 than TM, and that the best diagnostic performance was obtained by variance, with an AUC of 0.848. Variance is an important biological indicator for assessing tumor heterogeneity, reflecting the variation in tumor image grayscale and the discrete degree of lesion characteristics. Higher variance reflects a greater deviation from the mean value in the lesion, indicating non-uniform density and greater heterogeneity, as well as significantly increased cell proliferation activity in the tumor tissue (17, 18). Bohara M et al. (17) demonstrated that the variance extracted from ADC histogram parameters can effectively differentiate between high-grade meningioma from low-grade meningioma, and is significantly positively correlated with the cell proliferation index. In this study, SFT exhibited higher variance, and a significant positive correlation was also observed between variance and the Ki-67 proliferation index, which is consistent with the results of previous studies. This may be explained by the greater tumor heterogeneity of SFT, active tumor cell proliferation, and heterogeneous distribution of tissue components within the tumor.

The percentiles of ADC are also essential quantitative parameters for assessing tumor heterogeneity, which is closely related to tumor biological behavior and tumor cell proliferative activity. High-malignancy tumors exhibit greater heterogeneity, with active tumor cell proliferation and a significant increase in cell number and arrangement density. The densely arranged tumor cells restrict the activity of water, reflected as a decrease in ADC values (18). Chen T et al. (8) compared the differences between ADC of intracranial SFT and TM and found that ADC values are potential biomarkers for distinguishing between these two tumors. In our study, AP1 and AP10 were significantly lower for SFT than for TM, while the mean, AP50, AP90, and AP99 showed no significant differences. This may be because the lower-level percentiles of ADC represent the most active and densely proliferating areas within the tumor, thereby providing a more accurate and objective assessment of tumor heterogeneity. Liu X et al. (19) showed that lower-level percentiles of ADC are the reliable parameter for differentiating intracranial SFT from atypical meningioma. Similar results have also appeared in another study, which used percentiles of ADC to distinguish intracranial SFT from angiomatous meningioma (20).

We also found that the expression level of the Ki-67 proliferation index was significantly higher in the SFT group than in the TM group, with AP1 exhibiting the strongest correlation with the Ki-67 proliferation index among the percentiles of ADC. The Ki-67 proliferation index is a pathological biomarker of proliferative cells activity, and tumors with active cell proliferation tend to exhibit an increased Ki-67 proliferation index (21), whereas the ADC value is an imaging marker of proliferation cell activity, and tumors with active cell proliferation tend to exhibit decreased ADC value (18). The negative correlation observed between the Ki-67 proliferation index and ADC values further suggests that percentiles of ADC, especially lower-level percentiles of ADC, are reliable imaging biomarkers for the quantitative assessment of brain tumor heterogeneity. Yang H et al. (2) used ADC histogram parameters for grading intracranial SFT and explored the relationship between these parameters and the Ki-67 proliferation index. The result showed that lower-level percentiles of ADC not only yielded the best grading performance, but also exhibit the strongest correlation with the Ki-67 proliferation index, which is consistent with our study. Given the above, lower-level percentiles of ADC should therefore be given more attention when using ADC histogram analysis to evaluate brain tumors.

Skewness and kurtosis reflect the degree of asymmetry and peak in the distribution of ADC values, respectively, and both of them are important reference indicators for assessing tumor heterogeneity (22–24). Larger values of skewness and kurtosis tend to represent tumors with greater heterogeneity (22, 23). Baek HJ et al. (22) showed that skewness and kurtosis could provide an effective assessment of early treatment response in patients with glioblastoma. In this study, although the kurtosis did not show a significant difference between the SFT and TM groups, the kurtosis of SFT was numerically greater than that of TM, which may indicate greater heterogeneity of SFT. However, we also noted that the skewness between the two groups of tumors was similar and could not provide a valid distinction between SFT and TM. The reasons for such inconsistent results with previous literature are difficult to explain and may be related to differences in scanning parameters, sample size, and histogram analysis software.

Our study has a few limitations. First, as a single-center retrospective study, selection bias was inevitable. Second, manual sketching of the ROI for ADC histogram analysis was relatively time-consuming. Third, only the ADC images were included in the histogram analysis. Further studies with multiple centers and larger sample sizes are therefore required, and automatic segmentation techniques should be actively applied to the histogram analysis.

In conclusion, our study demonstrated that whole-volume ADC histogram analysis is a feasible tool for non-invasive preoperatively distinguishing intracranial SFT and TM, with the variance being the most promising prospective parameter, which may be helpful for clinical treatment decisions.

## Data availability statement

The raw data supporting the conclusions of this article will be made available by the authors, without undue reservation.

## Ethics statement

The studies involving human participants were reviewed and approved by Ethics Committee of Lanzhou University Second Hospital. Written informed consent for participation was not required for this study in accordance with the national legislation and the institutional requirements.

## Author contributions

GW: conception and design, data collection, data analysis, drafting of manuscript, major revisions, approved of submission on behalf of all authors. JZ: data analysis, drafting of manuscript, major revisions, article revision suggestions. All authors contributed to the article and approved the submitted version.

## References

[1] Kinslow CJ Bruce SS Rae AI Sheth SA McKhann GM Sisti MB. Solitary-fibrous tumor/hemangiopericytoma of the central nervous system: a population-based study. J Neurooncol (2018) 138(1):173–82. doi: 10.1007/s11060-018-2787-7 29427152

[2] Yang H Liu X Jiang J Zhou J. Apparent diffusion coefficient histogram analysis to preoperative evaluate intracranial solitary fibrous tumor: relationship to ki-67 proliferation index. Clin Neurol Neurosurg (2022) 220:107364. doi: 10.1016/j.clineuro.2022.107364 35872434

[3] Louis DN Perry A Wesseling P Brat DJ Cree IA Figarella-Branger D. The 2021 WHO classification of tumors of the central nervous system: a summary. Neuro Oncol (2021) 23(8):1231–51. doi: 10.1093/neuonc/noab106 PMC8328013 34185076

[4] Ma XJ Zhang GJ Wang W Li D Wu Z Zhang JT. Proposed treatment for intracranial transitional meningioma: a single-center series of 298 cases. World Neurosurg (2019) 127:e280–e7. doi: 10.1016/j.wneu.2019.03.104 30902770

[5] Zhang J Zhang G Cao Y Ren J Zhao Z Han T. A magnetic resonance imaging-based radiomic model for the noninvasive preoperative differentiation between transitional and atypical meningiomas. Front Oncol (2022) 12:811767. doi: 10.3389/fonc.2022.811767 35127543 PMC8815760

[6] Shin DW Kim JH Chong S Song SW Kim YH Cho YH. Intracranial solitary fibrous tumor/hemangiopericytoma: tumor reclassification and assessment of treatment outcome via the 2016 WHO classification. J Neurooncol (2021) 154(2):171–8. doi: 10.1007/s11060-021-03733-7 34417710

[7] Sung KS Moon JH Kim EH Kang SG Kim SH Suh CO. Solitary fibrous tumor/hemangiopericytoma: treatment results based on the 2016 WHO classification. J Neurosurg (2018), 1–8. doi: 10.3171/2017.9.JNS171057 29521591

[8] Chen T Jiang B Zheng Y She D Zhang H Xing Z. Differentiating intracranial solitary fibrous tumor/hemangiopericytoma from meningioma using diffusion-weighted imaging and susceptibility-weighted imaging. Neuroradiology (2020) 62(2):175–84. doi: 10.1007/s00234-019-02307-9 31673748

[9] Liu X Wang Y Wei J Li S Xue C Deng J. Role of diffusion-weighted imaging in differentiating angiomatous meningioma from atypical meningioma. Clin Neurol Neurosurg (2022) 221:107406. doi: 10.1016/j.clineuro.2022.107406 35932585

[10] Xianwang L Lei H Hong L Juan D Shenglin L Caiqiang X. Apparent diffusion coefficient to evaluate adult intracranial ependymomas: relationship to ki-67 proliferation index. J Neuroimaging (2021) 31(1):132–6. doi: 10.1111/jon.12789 32961009

[11] Surov A Gottschling S Mawrin C Prell J Spielmann RP Wienke A. Diffusion-weighted imaging in meningioma: prediction of tumor grade and association with histopathological parameters. Transl Oncol (2015) 8(6):517–23. doi: 10.1016/j.tranon.2015.11.012 PMC4700293 26692534

[12] Cao T Jiang R Zheng L Zhang R Chen X Wang Z. T1 and ADC histogram parameters may be an *in vivo* biomarker for predicting the grade, subtype, and proliferative activity of meningioma. Eur Radiol (2022) 33(1):258–269. doi: 10.1007/s00330-022-09026-5 35953734

[13] Xue C Liu S Deng J Liu X Li S Zhang P. Apparent diffusion coefficient histogram analysis for the preoperative evaluation of ki-67 expression in pituitary macroadenoma. Clin Neuroradiol (2022) 32(1):269–76. doi: 10.1007/s00062-021-01134-x 35029726

[14] Liu X Huang X Han T Li S Xue C Deng J. Discrimination between microcystic meningioma and atypical meningioma using whole-lesion apparent diffusion coefficient histogram analysis. Clin Radiol (2022) 77(11):864–9. doi: 10.1016/j.crad.2022.07.004 36030110

[15] Just N. Improving tumour heterogeneity MRI assessment with histograms. Br J Cancer (2014) 111(12):2205–13. doi: 10.1038/bjc.2014.512 PMC4264439 25268373

[16] Zhao L Liang M Yang Y Zhao XM Zhang HM. Histogram models based on intravoxel incoherent motion diffusion-weighted imaging to predict nodal staging of rectal cancer. Eur J Of Radiol (2021) 142:109869. doi: 10.1016/j.ejrad.2021.109869 34303149

[17] Bohara M Nakajo M Kamimura K Yoneyama T Fukukura Y Kiyao Y. Histological grade of meningioma: prediction by intravoxel incoherent motion histogram parameters. Acad Radiol (2020) 27(3):342–53. doi: 10.1016/j.acra.2019.04.012 31151902

[18] Gihr GA Horvath-Rizea D Hekeler E Ganslandt O Henkes H Hoffmann KT. Histogram analysis of diffusion weighted imaging in low-grade gliomas: *in vivo* characterization of tumor architecture and corresponding neuropathology. Front Oncol (2020) 10:206. doi: 10.3389/fonc.2020.00206 32158691 PMC7051987

[19] Liu X Deng J Sun Q Xue C Li S Zhou Q. Differentiation of intracranial solitary fibrous tumor/hemangiopericytoma from atypical meningioma using apparent diffusion coefficient histogram analysis. Neurosurg Rev (2022) 45(3):2449–56. doi: 10.1007/s10143-022-01771-x 35303202

[20] He W Xiao X Li X Guo Y Guo L Liu X. Whole-tumor histogram analysis of apparent diffusion coefficient in differentiating intracranial solitary fibrous tumor/hemangiopericytoma from angiomatous meningioma. Eur J Radiol (2019) 112:186–91. doi: 10.1016/j.ejrad.2019.01.023 30777209

[21] Sun X Kaufman PD. Ki-67: more than a proliferation marker. Chromosoma (2018) 127(2):175–86. doi: 10.1007/s00412-018-0659-8 PMC5945335 29322240

[22] Baek HJ Kim HS Kim N Choi YJ Kim YJ. Percent change of perfusion skewness and kurtosis: a potential imaging biomarker for early treatment response in patients with newly diagnosed glioblastomas. Radiology (2012) 264(3):834–43. doi: 10.1148/radiol.12112120 22771885

[23] Ren JL Yuan Y Li XX Shi YQ Tao XF. Histogram analysis of apparent diffusion coefficient maps in the prognosis of patients with locally advanced head and neck squamous cell carcinoma: comparison of different region of interest selection methods. Eur J Radiol (2018) 106:7–13. doi: 10.1016/j.ejrad.2018.07.004 30150054

[24] Raab P Banan R Akbarian A Esmaeilzadeh M Samii M Samii A. Differences in the MRI signature and ADC values of diffuse midline gliomas with H3 K27M mutation compared to midline glioblastomas. Cancers (Basel) (2022) 14(6). doi: 10.3390/cancers14061397 PMC8946584 35326549

